# Effects of population structure on pollen flow, clonality rates and reproductive success in fragmented *Serapias lingua* populations

**DOI:** 10.1186/s12870-015-0600-8

**Published:** 2015-09-16

**Authors:** Giuseppe Pellegrino, Francesca Bellusci, Anna Maria Palermo

**Affiliations:** Dept. of Biology, Ecology and Earth Sciences, University of Calabria, I-87036 Rende, (CS) Italy

## Abstract

**Background:**

Fragmentation of habitats by roads, railroads, fields, buildings and other human activities can affect population size, pollination success, sexual and asexual reproduction specially in plants showing pollinator limitation, such as Mediterranean orchids. In this study, we assessed pollen flow, selfing rates, vegetative reproduction and female reproductive success and their correlations with habitat characters in nine fragmented subpopulations of *Serapias lingua*.

To improve understanding of population structure effects on plant biology, we examined genetic differentiation among populations, pollen flow, selfing rates and clonal reproduction using nuclear microsatellite markers.

**Results:**

Smaller populations showed a significant heterozygote deficit occurred at all five nuclear microsatellite loci, the coefficient of genetic differentiation among populations was 0.053 and pairwise F_ST_ was significantly correlated with the geographical distance between populations. Paternity analysis of seeds showed that most pollen flow occurred within a population and there was a positive correlation between percentage of received pollen and distance between populations.

The fruit production rate varied between 5.10 % and 20.30 % and increased with increasing population size, while the percentage of viable seeds (78-85 %) did not differ significantly among populations. The extent of clonality together with the clonal and sexual reproductive strategies varied greatly among the nine populations and correlated with the habitats where they occur. The small, isolated populations tended to have high clonal diversity and low fruit production, whereas the large populations with little disturbance were prone to have reductions in clonal growth and increased sexual reproduction.

**Conclusions:**

We found that clonality offers an advantage in small and isolated populations of *S. lingua*, where clones may have a greater ability to persist than sexually reproducing individuals.

## Background

Fragmentation of plant populations, the process by which formerly continuous populations turn into patches of different sizes, isolated from each other, may have distinctive effects on populations: (1) affecting reproductive success, (2) altering patterns of pollen-mediated gene flow (pollen flow) and (3) affecting self-pollination and vegetative propagation. Although many plant populations are naturally isolated and small, populations of numerous plant species have become more isolated as a result of the recent anthropogenic fragmentation of habitats by roads, railroads, fields, buildings and other human activities [1, 2].

Fragmentation and the abundance of a plant species can have striking effects on the visitation rate and floral constancy of its pollinators, with potentially major impacts on the plant's reproductive success, reducing the abundance and species richness of pollinators, altering their foraging behaviour and limiting pollinator movement among populations [3, 4]. Thus, plants receive fewer flower visits suffering pollen limitation and reduction in reproductive success. Studies of local population density and size clearly show that pollination and reproductive success decrease in sparse and small populations [5]. Reductions in reproductive success due to reduced insect movements are particularly strong for plants which show a high degree of dependence on their pollinator mutualism (i.e. pollinator limitation) for fruit production [6], such as Mediterranean deceptive orchids [7].

Sexual reproduction is predominantly pollinator dependent, even if it may sometimes be successfully guaranteed by asexual reproduction or self-pollination. Self-pollinating populations are more likely to establish in habitats where pollinators appear to be scarce, in which population size is small [8], and in environments with limited opportunity for outcrossing [9].

The complex flower structures and pollination strategies of orchids are the best-documented examples of selection for outcrossing in flowering plants to avoid inbreeding. However, auto-pollinating orchids are relatively frequent in geographically isolated and/or pollinator-scarce environments such as higher latitudes/elevations, coastal areas and islands [10, 11], supporting the ‘reproductive assurance’ hypothesis in which selection favours increased self-pollination to ensure the persistence of populations in situations in which pollinator service strongly limits reproduction [12]. Approximately 20 % of terrestrial orchid species in which the pollination system has been investigated are capable of auto-pollination [11, 13], suggesting that autopollination is indeed common in Orchidaceae [14].

In the plant kingdom reproduction can be assured by vegetative reproduction, a typical asexual reproduction whereby new individuals are formed without the production of seeds, including the formation of new plants out of rhizomes, bulbs or tubers. Vegetative propagation leads to a clonal structure in which one clone (genet) may consist of several individuals (ramets). The most obvious genetic signature of vegetative propagation in a population is the presence of repeated multilocus genotypes (MLGs) and, as a consequence, heterozygosity and allelic diversity at each locus are expected to increase [15]. Many orchid species have the capacity for vegetative propagation which can represent the prevalent pattern of population maintenance. There are several patterns of vegetative reproduction in orchids, varying between species possessing different life forms [16]. The most widespread pattern of vegetative multiplication in orchids is the formation and germination of two or more buds, including dormant ones, on axial organs such as rhizomes, creeping shoots and shoot tubers [17]. The daughter shoots are connected with the maternal ones for a long time. The daughter shoots in orchids with shoot rhizomes or bulbotubers (*Anacamptis*, *Dactylorhiza*, *Orchis*, *Ophrys*, *Serapias*, etc.) separate most rapidly, after 1–2 years [18]. Among orchids we can distinguish those with obligate vegetative propagation, those with facultative vegetative propagation, which includes short-rhizome and most tuberoidous orchids, and those with vegetative propagation occurring in exceptional cases [16].

An explicit method to clarify and quantify the direction of pollen flow between populations and to verify the presence of spontaneous self pollination or vegetative reproduction is the molecular analysis of plants and paternity analysis of seeds collected from known mothers to determine the origin of the pollen that fertilized the ovules.

In this study, we assessed pollen flow, selfing rates, vegetative reproduction and female reproductive success in nine fragmented subpopulations of an orchid species, *Serapias lingua*. This species dependent upon insect pollinators to ensure its reproduction, is self-compatible and able to vegetatively reproduce [19] and thus, is suitable for investigating the effects of population fragmentation on gene flow, selfing/clonality rates and reproductive success.

More specifically, we aimed at (1) determining the genetic population structure to quantify clonality rates; (2) examining fruit production rates in the studied populations to obtain estimates of female reproductive success; and (3) examining a paternity analysis of seeds collected from the plants.

## Methods

### Study species

The genus *Serapias* L. is distributed throughout the Mediterranean region with its centre of diversity in southern Italy and on the Greek islands [20].

*Serapias lingua* (tongue orchid) is a short-lived tuberous orchid and a tetraploid species [21]. It has dull-coloured flowers of uniform structure: the all three sepals and the hypochile (the proximal part of the lip) form a hood (tubular corolla), a unique shiny, more or less round callosity, is present at the base of the hypochile, the epichile (the distal part of the lip) is generally inclined downwards. The petals and lip are characterized by conical epidermal papillae and two types of trichome with secretory apical cells [22]. It is a widespread species, mainly distributed in the Mediterranean-Atlantic countries (Portugal, Spain, France, Italy, Balkans, Greece), but reaching western North Africa (Morocco, Tunisia). It grows in arid or wet meadows, abandoned agricultural soils, garigue and bushy environments up to 1200 m a.s.l. [23]. Recent molecular analysis strongly supports a natural split of *S. lingua* into a subgroup strictly related to *S. gregaria* and *S. olbia*, two rare endemics of the Var and Maritime Alps regions [24].

In the last years the pollination strategy of *S. lingua* has received more attention, and preliminary observations indicate that *Ceratina cucurbitina* males are the main pollinators [14, 25]. While other *Serapias* species offer insects a floral tube in which to rest or sleep (shelter imitation strategy), *S. lingua* seems to have evolved to sexually deceive pollinators, analogous to what is observed in *Ophrys* orchids [26], a phenomenon also supported by the finding of large amounts of alkanes and alkenes in its floral odour extracts [27, 28].

### Study area and measures of population size and density

The research site is located in southern Italy (Calabria region). It covers approximately 700 ha and consists of calcareous, dry grasslands (Festuco-Brometalia); *Spartium junceum* L., *Cytisus sessilifolius* L. and *Cistus incanus L.* are frequent shrubs and *Festuca circummediterranea* Patzke*, Bromus erectus* Huds. and *Dactylis glomerata* L. are the dominant herbs*.*

**Fig. 1:**
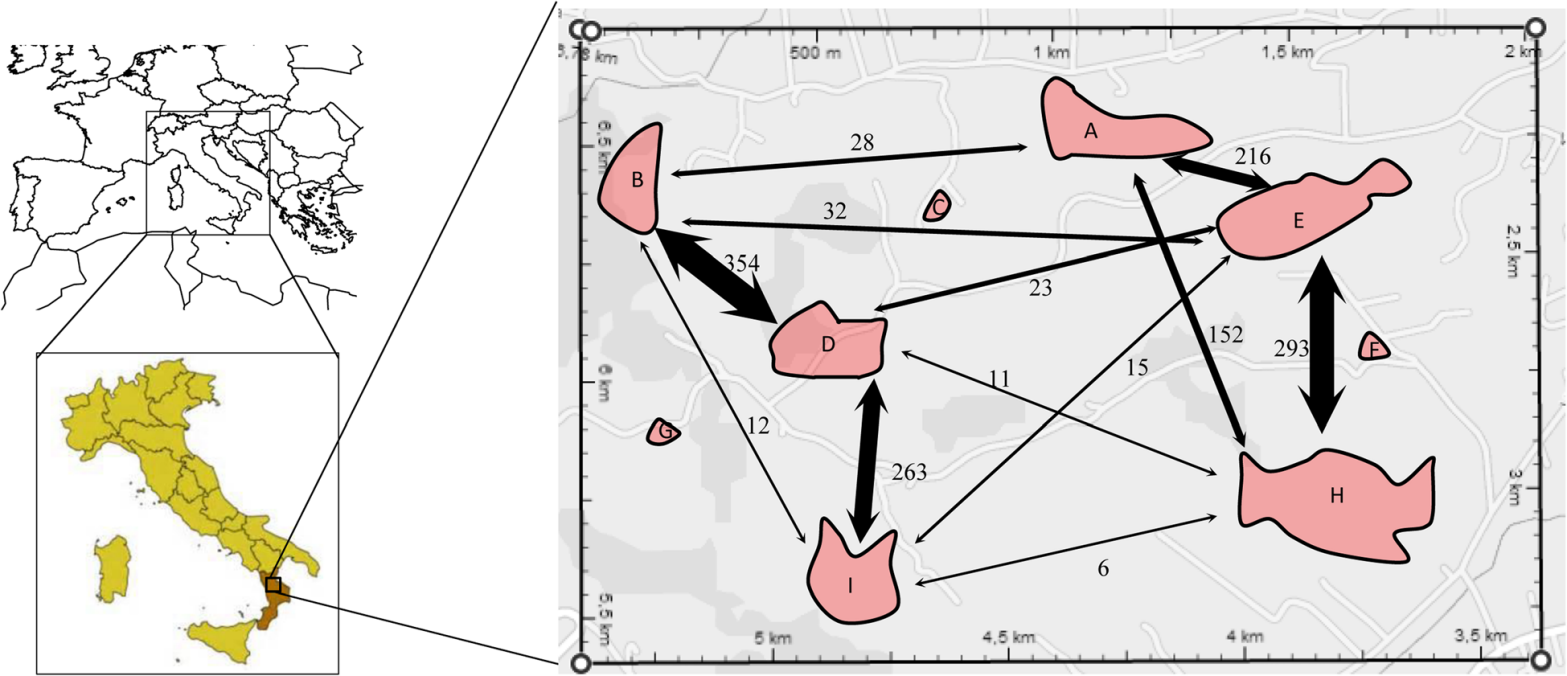
Spatial distribution of *Serapias lingua* populations. Red areas indicate the nine populations defined by this study. Arrows represent pollen flow and the numbers by the arrows indicate the numbers of pollen migration events. Figure was created by G. Pellegrino (the first author)

*Serapias lingua* grows over the entire area, forming populations of a few to thousands of individuals. We define ‘a population’ here as a group of *S. lingua* individuals in a discrete area, each of which is separated from a neighbouring population by at least 300 m (Fig. 1). A total of 9 populations were identified; three (C, F, G) are found in a highly anthropic landscape context enclosed by busy roads and their intersections, while the remaining six (A, B, D, E, H, I) are non-anthropic (natural) populations. No other population is present in or around the study area and the nearest population outside the study area is about 5 km north of population A.

In Spring 2014 the population size (i.e. the total number of individuals in a specific area) and population density (i.e. the population size divided by total area) was determined for each population. For population size, we individually marked and counted the number of all (flowering and vegetative) individuals in the three smaller populations (C, F, G), while within each other populations we marked and counted the number of individuals in five selected square grid (10 by 10 m size) separated by 30–50 m. The measurements resulting from the five plots for each population were grouped and used to calculated population size. For population density, we calculated the area of the population (in square metres) identifying the boundaries of each population using the outermost individuals (Table 1). Voucher specimens were deposited at the herbarium at the University of Calabria (CLU).

**Table 1:** Population size and density, fruit production rate, percentage of viable seeds, immigration rate by pollen per population

Population	Pop area (in square meters)	Pop size	Pop density	Fruit set (%)	Viable seeds (%)	Immigration rate by pollen (%)	Pollen source population								
							A	B	C	D	E	F	G	H	I
A	3578.25	~2800	0.78	13.58	82.78 ± 3.73	28.68	553	6			114			112	
B	2540.20	~2000	0.79	20.30	79.85 ± 2.44	32.02	22	571		203	24				
C	64.20	302	4.70	5.20	78.55 ± 2.13	9.38		1	29	1					
D	3451.22	~3000	0.87	14.23	81.21 ± 2.86	28.34		151		610	8		1		81
E	2962.40	~2500	0.84	15.60	85.35 ± 3.83	27.49	102	8		15	565			84	6
F	55.80	321	5.75	5.50	81.21 ± 3.27	11.11					2	32		2	
G	65.54	284	4.31	5.10	82.24 ± 2.33	7.14				2			26		
H	4585.30	~3200	0.70	14.68	82.54 ± 3.66	30.53	40			11	209			609	6
I	2542.60	~2200	0.86	16.75	79.65 ± 2.05	28.64		12		182	9	1			535

### Measures of reproductive success

To test natural reproductive success, in the three smaller populations and in five square grid for each of the remaining six populations, the number of flowers that produced fruits was counted and the fruit set was determined as the average of ratios (number of produced fruits/number of available flowers) over the examined plants. To ascertain the presence of viable embryos, at least 1000 seeds from each fruit were removed from the centre of the capsule and observed under an optical microscope (100x). Seeds were assigned to two categories (viable and unviable seeds) due to the presence or absence of viable embryos. The seed set [(the number of filled seeds in sampled fruits/the number of observed seeds) × 100] were calculated for every fruit.

In addition, in each population five individuals with unopened flowers were bagged with a fine-meshed cloth to exclude pollinators to test for spontaneous autogamy. In June, the number of produced fruits was counted, and the ratio between the number of fruits/treated flowers was determined.

### DNA extraction and microsatellite genotyping

One leaf from each individual in the three smaller populations and from each individual in the five selected areas of other six populations was sampled and stored in silica gel for subsequent DNA extraction and microsatellite (Short Sequence Repeat, SSR) genotyping. Genomic DNA was extracted using a slight modification of the CTAB (cetyltrimethyl ammonium bromide) protocol of Doyle and Doyle [29]. Approx. 0.5 g of each leaf were separately pestled in a 2 ml-Eppendorf vial using 500 μL of standard CTAB buffer, incubated at 60 °C for 30 min, extracted twice by adding 500 μL chloroform-isoamyl alcohol (24:1), precipitated with isopropanol and washed with 250 μL of ethanol 70 %. The DNA was re-suspended in 50 μL of distilled water.

To characterize the genetic structure of each population and genotype, we performed microsatellite genotyping on all the adult plants using five nuclear microsatellite loci previously isolated and tested on *Serapias* sp. [19, 30]. All PCR reactions of 100 μl final volume contained 40 ng of genomic DNA, 100 μM of each dNTP, 0.3 μM of each primer, 2 units of Taq polymerase, 2 μM MgCl_2_ and 10 μl of reaction buffer. The amplification conditions were: 1 cycle of 94 °C for 3 min;30 cycles 30 s at 94 °C, 45 s at the locus specific annealing temperature (55 or 58 °C), and 30 s at 72 °C using a Perkin Elmer thermal cycler. One of the PCR primers for each locus was labeled with fluorescent dye (FAM, TET). Labelled PCR products were run together with the internal size standard GeneScan ROX400 on an ABI 3110 (Perkin Elmer, Biosystems), and individuals were genotyped using Genescan Analysis software and Genotyper software (Perkin Elmer, Biosystems).

### Clonality rates

Multilocus genotypes (MLGs) were assigned manually. Because individuals with the same MLG found in populations with both sexual and vegetative reproduction can be either ramets of the same genet or derive by chance from distinct events of sexual reproduction, we used the program GIMLET 1.3.2 [31] to estimate the probability that two individuals, randomly sampled from a population, shared the same MLG by chance (probability of identity: PI).

Two different genotypic diversity indexes were calculated. The first measure was G/N, the ratio between the number of MLGs and the total number of individuals in a population [32]. Values of this index vary from zero (strict clonality) in which all individuals share the same MLG, to one (sexual reproduction) in which each individual has a distinct MLG. The second measure was MLG diversity (D_G_) [33] which measures the probability that two individuals randomly selected from a population of N individuals will have different MLGs. Similar to the first measure, D_G_ ranges from zero indicating that there is only one dominant clone, to one suggesting that every individual has a different genotype.

### Genetic variability

Population genetic analyses were based on a ‘corrected’ dataset in which all individuals with the same MLG were considered as ramets of a single genet. For nSSRs, the number of alleles, number of alleles per locus (N_a_) and per population (N_ap_) [34], observed heterozygosity (H_O_), gene diversity (H_E_) [35], and fixation index (F_IS_ = 1 – H_O_/H_E_) were calculated for each locus and each population using FSTAT version 2.9.3.2 [36]. Departures from Hardy–Weinberg equilibrium at each locus and linkage disequilibrium between loci were tested by an exact test using a Markov chain method implemented in GENEPOP version 4.0 [37], with Bonferroni corrections. H_T_ and H_S_ [35], and F_ST_ [38] were estimated using FSTAT. H_T_ is the gene diversity in the total population, H_S_ is the average gene diversity within populations, and F_ST_ is the coefficient of genetic differentiation among populations under an infinite allele model. Pairwise F_ST_ values were tested for significance by permuting genotypes among populations. To test for the presence of isolation by distance, a Mantel test between population-pairwise geographic distance and F_ST_/(1 – F_ST_) was applied [37]. Null allele (alleles that did not give a polymerase chain reaction product) frequencies were estimated using the maximum-likelihood (ML) estimator based on the EM algorithm and implemented by default in GENEPOP 4.0 [37]. Based on microsatellite allele frequencies, recent population bottlenecks were checked by BOTTLENECK [39], employing the Two Phase Mutation model (TPM) with a 95 % Stepwise Mutation Model (SMM) and 5 % multistep mutations. Significance was assessed using the Wilcoxon test. The bottleneck program [40] was used as an alternative measure of genetic bottlenecks to test for excess gene diversity relative to that expected under mutation-drift equilibrium. The heterozygosity excess method exploits the fact that allele diversity is reduced faster than heterozygosity during a bottleneck, because rare alleles are lost rapidly and have little effect on heterozygosity, thus producing a transient excess in heterozygosity relative to that expected in a population of constant size with the same number of alleles [39].

### Paternity assignment

Microsatellite profiles for each fruit were also determined to ascertain if fruit developed by plants in each population could have been produced by pollen transferred by individuals of the same population or different donors. In June, capsules were collected and seeds in the central part were used for molecular analysis. Seeds were observed under a binocular microscope and approx. 50 viable seeds (which means seeds with an embryo) from each capsule were collected and transferred into single 2 ml-Eppendorfs to extract their DNA. Nuclear microsatellite loci were amplified and analyzed following the protocol described above. Paternity analysis was performed by a likelihood-based approach based on multilocus genotypes for all adult genets and offspring using CERVUS version 2.0 [41]. In this study, the simulation parameters required by the program were set as follows: 10 000 cycles, 4956 candidate parents (= all fruits collected across the study population), 0.99 as the proportion of candidate parents sampled, and 1.00 and 0.001 as the proportions of loci typed and mistyped, respectively.

According to the assigned paternity data, we categorized the fruit as derived from selfing, outcrossing within the study area, and outcrossing with a paternal parent that was not present in the study area. We defined the selfing rate as the number of selfed fruits divided by the number of examined fruits from each population.

## Results

### Population size and density

The stands differed in population size, ranging from 284 to ~3200 individuals, in population density (0.70–5.75 individuals/m^2^) (Table 1) and degree of isolation (the distance between *S. lingua* populations ranged from 300 m to 2.5 km). Three populations (C, F, G) showed significantly lower values of population size and higher values of population density than the other six populations, such as they had lower population areas (Table 1).

### Reproductive success

Significant differences were detected among the populations in their fruit production rate. Indeed, the populations differed significantly in their fruit sets, which varied from 5.10 % to 20.30 % and was 14.53 % for the nine populations on average. More specifically, the three smallest populations in term of population size (C, F, G) showed lower values than the other populations, which showed values four times higher (Table 1). In contrast, the populations did not differ significantly in their percentage of viable seeds, which varied from 78.55 (±2.13) for population C to 85.35 (±3.83) for population E (Table 1). The best explanation for the variation in the fruit production rate is the positive correlation between fruit set and population size. Indeed, the estimated parameter for the population size was positive, suggesting that larger populations have higher outcrossing rates.

None of the 45 individuals (five per population) bagged with a fine-meshed cloth to exclude pollinators showed any spontaneous autogamy.

### Presence and extent of clonal propagation

All populations were affected by different levels of clonality. The population with the lowest G/N ratio was C (0.067), and slightly higher values were shown by the other two (F and G) small populations (Table 2). Higher G/N values were found in the other populations, ranging from 0.812 (population A) to 0.892 (population H). Similar results were found for multilocus genotype diversity (D_G_), which ranged from close to zero (population C) to 0.794 (population H), with a mean value of 0.215 (Table 2).

**Table 2:** Measures of clonal propagation: ratio between the number of multilocus genotypes and the total number of individuals (G/N), and multilocus genotype diversity (D_G_) in nine populations of *S. lingua*

Population	G/N	D_G_
A	0.812	0.721
B	0.885	0.748
C	0.067	0.038
D	0.862	0.740
E	0.854	0.725
F	0.085	0.040
G	0.088	0.041
H	0.892	0.794
I	0.886	0.784
mean	0.603	0.515

### Genetic diversity and differentiation among populations

PCR products were successfully obtained from all examined individuals, their fragment lengths fit into the predicted size ranges, and all examined loci were polymorphic across the nine populations. No significant linkage disequilibrium between loci was observed for any population, so all loci were used for further analyses.

**Fig. 2:**
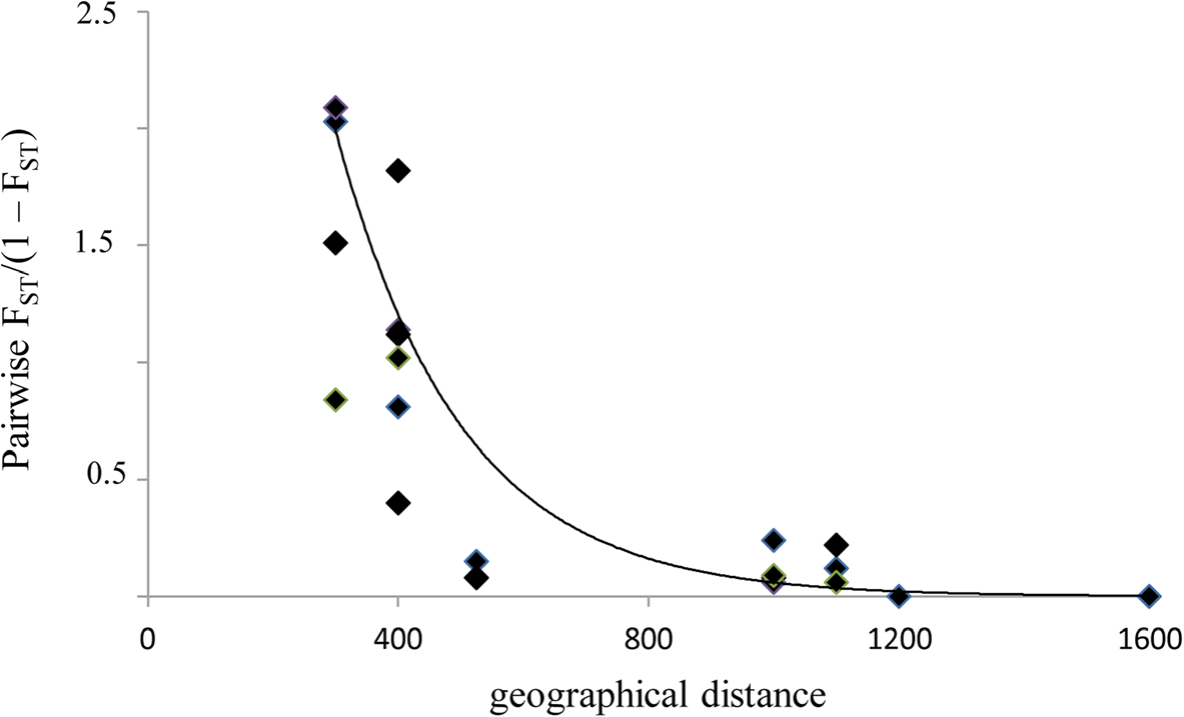
The correlation between pairwise F_ST_/(1 – F_ST_) and geographical distance

The total number of alleles per population ranged between 4 and 15 (average 9.6 alleles) and the number of alleles per locus ranged between 8 and 20 (data not shown). Three populations (C, F, G) had a lower mean allele number per population than the other populations, and possessed all alleles exhibited by natural populations. Moreover, in anthropic populations the observed heterozygosity was much less than expected (H_O_ = 0.38-0.42;H_E_ = 0.52-0.60), while the other populations possessed higher heterozygosity (H_O_ ranging from 0.77 to 0.80) that was close to expected values (H_E_ ranging from 0.75 to 0.79) (Table 3). Inbreeding coefficients (F_IS_) calculated at each nSSR locus in each population (45 values) varied among populations. Six populations showed a low heterozygote excess ranging from F_IS_ = −0.02 (pop E) to F_IS_ = −0.12 (pop A), while three others showed a significant heterozygote deficit (F_IS_ = 0.22-0.28) at all five loci (Table 3). Few private alleles were found in each population. The coefficient of genetic differentiation among populations (F_ST_) was estimated to be 0.053 for nSSRs. Pairwise F_ST_/(1 – F_ST_) was significantly correlated with the geographical distance between populations for nSSRs (P < 0.05, Fig. 2).

**Table 3:** Measures of number of alleles per population (N_ap_), observed (H_O_) and exptected (H_E_) heterozygosity, and fixation index F_IS_ in nine populations of *S. lingua*

Population	N_ap_	H_O_	H_E_	F_IS_
A	15	0.784	0.774	−0.12
B	9	0.774	0.752	−0.04
C	4	0.418	0.594	0.25
D	10	0.789	0.755	−0.07
E	11	0.776	0.762	−0.02
F	6	0.422	0.524	0.28
G	5	0.382	0.516	0.22
H	14	0.782	0.789	−0.08
I	12	0.777	0.778	−0.04
Average	9.6	0.656	0.694	0.04

Bottleneck analysis revealed that three populations had a significantly higher observed gene diversity than expected under the 95 % Stepwise Mutation Model, while no deviation from mutation-drift equilibrium was found for any other population. In a population at mutation-drift equilibrium (i.e., the effective size has remained constant in the recent past), there is an approximately equal probability that a locus shows either a gene diversity excess or a gene diversity deficit. Populations that have experienced a recent reduction in their effective population size exhibit a correlative reduction in the number of alleles and gene diversity at polymorphic loci. But the number of alleles is reduced faster than the gene diversity. Thus, in a recently bottlenecked population, the observed gene diversity is higher than the expected equilibrium gene diversity computed from the observed number of alleles, under the assumption of a constant-size (equilibrium) population [42].

### Paternity assignment of seeds

In the paternity assignment experiments, 4967 fruits were obtained from 5176 plants in nine populations (Table 1). DNA extraction failed for 21 samples, but the paternity of the remaining 4956 was examined and identified at a 95 % confidence level. There was significant differentiation by the paternity test among populations in term of the percentage of immigration rate, which varied from 7.14 % (population G) to 32.02 % (population B). Indeed, in six populations (A, B, D, E, H and I) the pollen parents of approx 30 % of the fruit were located outside each population, and the remaining 70 % within the population, while in three populations (C, F, G) the pollen parents of ~90 % and ~10 % of the fruit were located within and outside each population, respectively. The mother plants of populations A, B, D, E, H and I received pollen widely from other populations. The maximum pollen dispersal distance within the whole population was 1100 m. Interestingly, there was a positive correlation between the percentage of received pollen and the distance between populations (Fig. 1). Indeed, greater gene flow occurred between the nearest populations, while gene flow was close to zero among the most distant populations. No fruits were produced by selfing.

## Discussion

### Population genetic structure

In this study analysis of microsatellite DNA variation in *Serapias* revealed clear and significant genetic differentiation among populations, suggesting different levels of gene flow between them.

In our investigations the number of alleles per locus (8–18) and the mean of 9.6 alleles per population are higher values than the alleles per locus (4–10) and alleles per population (3.6-5.6) detected by Pellegrino et al. [19, 43] in populations of other *Serapias* species (*S. parviflora*, *S. politisii* and *S. vomeracea*). But these values are similar to or slightly lower than those reported to date for other Mediterranean orchid genera, *Dactylorhiza* [44], *Gymnadenia* [45, 46], and *Ophrys* [47, 48].

The five markers included in this study showed medium levels of genetic variation (H_E_ ranging from 0.69 to 0.79, average 0.694) compared with other microsatellite studies on orchids [47].

The low value of genetic differentiation among populations (F_ST_=0.053) is due to the small geographic range of the *S. lingua* populations studied. Indeed, similar genetic differentiation values based on microsatellites have been reported in other small orchid populations of *Caladenia huegelii* [49] and *Gastrodia elata* [50], showing geographic distances of 150 and 250 km, respectively.

Patterns of population genetic diversity and viability may vary greatly across populations due to a multitude of possible variables [51]. Populations may lose most of their genetic diversity if they become very small and isolated [52]. Accordingly, we detected two distinct groups; first group formed by the three smallest *S. lingua* populations (C, F, G) showed a substantial deficit in genetic diversity, the largest difference between observed and expected heterozygosity, and higher values of inbreeding coefficients (F_IS_), while the second group formed by the other populations possessed observed heterozygosity close to expected heterozygosity values and lower values of inbreeding coefficients (Table 3). The genetic poorness of smaller populations often derives from limited connections to other populations [53].

### Paternity test and gene flow

Data from the paternity test of seeds showed that there were high frequencies of short-distance and low frequencies of long-distance pollen dispersal events. In the study populations, greater gene flow occurred between the nearest populations (distance from 300 to 500 m), while the rate of gene flow decreased in populations farther from each other (distance from 1000 to 1500 m) and there was little or no inter-population gene flow between the three smallest and most isolated populations (Fig. 2). In addition, these three populations showed that the flowers were pollinated in 90 % of cases by the pollen of the same population and only 10 % by pollen from other populations, which in contrast showed a greater flow of pollen input. Pollination events between populations increased with the geographical separation of the populations, suggesting that most movements of pollinators occur within populations. This is probably a consequence of inadequate pollinator visitation to small populations, resulting in strong gene flow limitation [2, 54]. The greater flow of pollen between the nearest populations is in agreement with the behaviour of pollinators. Indeed, recent work based on the capture and recapture of pollinating insects showed that the average distance travelled by pollinators was 300 m, and only a few insects were recaptured at distances of approximately 1000 m [55]. But this does not explain the lower pollen flow from outside the smaller populations in comparison with the larger populations, independent of the distance between the populations. Probably, there are other factors that determine this reduction. For example, one factor may be the population size, since the examined populations showed that proportions of out-of-plot pollen flow were positively correlated with the number of adult plants within the population. Larger populations of plants are likely to be more attractive to pollinators, resulting in higher visitation rates, whereas small fragmented populations may be less attractive [56]. In addition, a population with a longer perimeter will likely have more insects (i.e. pollinators) encounter it, resulting in increased pollination. Moreover, a higher population density can result in greater pollination between individuals in the same population or an increase in the selfing rate [57]. In our case, as the species is self-compatible, but not capable of producing fruits via spontaneous autogamy, the detected patterns can only be the result of active pollen transfer by pollinators, and thus the pollination success of *S. lingua* was significantly and positively related to population size. This is in accordance with the outcome of several studies on orchids that have already shown that gene flow is often positively affected by increasing population size [58]. In addition to the population size, our study indicated that the population density of flowering plants also affected pollinia removal, which increased when the local density decreased. This data is in apparent contrast with many previous papers on food-deceptive orchids, and in agreement with studies on sexually deceptive orchids. Indeed, Vandewoestijne et al. [59] showed that pollinator activity generally increased with decreasing population density in three *Ophrys* species, suggesting that pollinator availability, rather than pollinator learning, is the most limiting factor in successful pollination for sexually deceptive orchids. Moreover, in sexually deceptive orchids, insects rarely switch from one individual to another close individual immediately after the first attempted copulation, preferring to fly off at a greater distance from the first individual [60], suggesting that the apparent avoidance of multiple copulations within a small population will promote pollen flow over a greater distance [61].

### Sexual reproductive success and clonality rates

The results reported here showed that clonality represents a common reproductive strategy in all analysed populations, but clonality did not affect the different populations of *S. lingua* equally. Six larger *S. lingua* populations showed higher levels of clonality (DG = 0.71-0.79), for example, similar to those found in the endangered species *Cypripedium calceolus* (DG = 0.97; [62]), while the lowest clonal diversity (*G/N* index) and reduced heterozygosity (H_O_ = 0.38-0.42) in smaller populations, similar to those found in polish *Epipactis atrorubens* [63] and *Cephalantera rubra* populations [64], was a consequence of particularly intensive vegetative reproduction. According to our data, the C, F, and G populations showed a higher rate of clonality, while in other populations sexual strategies seemed to contribute more to reproduction. A hypothesis that may explain the pattern of clonality that we found in smaller populations is low sexual reproduction in these populations due to pollinator limitation, as evidenced by the small number of fruits produced. The balance between sex and clonal growth varies between and within species and is mainly driven by biotic and environmental factors [65]. Although vegetative propagation has ecological costs related to greater resource uptake, reduced pollen dispersal, or increased geitonogamous pollination [66], species showing higher rates of clonality have several potential ecological and evolutionary advantages. In our case, *S. lingua* can persist in small, isolated populations where conditions are not favourable for sexual reproduction, providing a form of reproductive assurance by guaranteeing the survival of the species in case of limited pollinator service [15]. Thus, the combination of the availability of pollinators and the fruit set related to population size characterizing each population and the distance between neighbouring populations of *S. lingua* can explain the different levels of clonal propagation we found in different populations. In particular, a higher rate of asexual reproduction was found in C, F, and G than in other populations, the former consisting of a few hundred individuals located in a restricted area (about 70 m^2^) closed to a crossroads, the latter comprising a thousand individuals in a larger area (~0.5 ha). Populations subjected to more environmental stress and fragmentation by roads, railroads, fields, buildings and other human activities show higher levels of clonality [15, 67].

## Conclusions

This study represents one of the few analyses of the effects of population structure on the pollen flow and clonal growth of a deceptive Mediterranean orchid. Population fragmentation is likely to reduce reproductive success due to reductions in population sizes and increases in the geographic distance between populations. We found that clonality offers an advantage in small and isolated populations of *S. lingua*, whereby clones may have a greater ability to persist than sexually reproducing individuals [61]. Since clonal growth is associated with a progressive reduction in genotypic diversity, sexual reproduction might be indispensable to the long-term success of a species and clonal growth may play an important role in prolonging the time to extinction when sex is reduced or absent.

## Abbreviations

CTAB: Cetyltrimethyl ammonium bromide
D_G_: Multilocus genotype diversity
F_IS_: Fixation index
F_ST_: Coefficient of genetic differentiation among populations
H_E_: Gene diversity
H_O_: Observed heterozygosity
H_S_: Average gene diversity within populations
H_T_: Gene diversity in the total population
ML: Maximum-likelihood
MLG: Multilocus genotypes
N_a_: Number of alleles per locus
N_ap_: Number of alleles per population
PI: Probability of identity
SMM: Stepwise mutation model
SSR: Short sequence repeat
TPM: Two phase mutation

## References

[1] Newman BJ, Ladd P, Brundrett M, Dixon KW (2013). Effects of habitat fragmentation on plant reproductive success and population viability at the landscape and habitat scale. Biol Conserv.

[2] Pellegrino G, Bellusci F (2014). The effects of human disturbance on the demography and reproductive success of *Serapias cordigera* (Orchidaceae). Bot J Linn Soc.

[3] Aguirre A, Dirzo R (2008). Effects of fragmentation on pollinator abundance and fruit set of an abundant understory palm in a Mexican tropical forest. Biol Conserv.

[4] Öckinger E, Dannestam Å, Smith HG (2009). The importance of fragmentation and habitat quality of urban grasslands for butterfly diversity. Landsc Urban Plan.

[5] Vranckx G, Jacquemyn H, Mergeay J, Cox K, Janssens P, Gielen BAS (2014). The effect of drought stress on heterozygosity–fitness correlations in pedunculate oak (*Quercus robur*). Ann Bot-London.

[6] Nayak KG, Davidar P (2010). Pollinator limitation and the effect of breeding systems on plant reproduction in forest fragments. Acta Oecol.

[7] Tremblay R, Ackerman JD, Zimmerman JK, Calvo RN (2005). Variation in sexual reproduction in orchids and its evolutionary consequences: a spasmodic journey to diversification. Biol J Linn Soc.

[8] Busch JW (2005). The evolution of self-compatibility in geographically peripheral populations of *Leavenworthia alabamica* (Brassicaceae). Am J Bot.

[9] Cheptou PO, Avendano V, Lyz G (2006). Pollination processes and the Allee effect in highly fragmented populations: consequences for the mating system in urban environments. New Phytol.

[10] Gamish A, Fischer GA, Comes HP (2014). Recurrent polymorphic mating type variation in Madagascan *Bulbophyllum* species (Orchidaceae) exemplifies a high incidence of auto-pollination in tropical orchids. Bot J Linn Soc.

[11] Zhou X, Lin H, Fan X-L, Gao J-Y (2012). Autonomous self-pollination and insect visitation in a saprophytic orchid, *Epipogium roseum* (D.Don) Lindl. Aust J Bot.

[12] Theologidis I, Chelo IM, Goy C, Teotònio H (2014). Reproductive assurance drives transitions to self-fertilization in experimental Caenorhabditis elegans. BMC Biol.

[13] Peter CI, Johnson SD (2009). Reproductive biology of *Acrolophia cochlearis* (Orchidaceae): estimating rates of cross-pollination in epidendroid orchids. Ann Bot-London.

[14] van der Cingel NA. An atlas of orchid pollination: European orchids. London, UK: CRC Press; 2001.

[15] Meloni M, Reid A, Caujapé-Castells J, Marrero Á, Fernández-Palacios JM, Mesa-Coelo RA (2013). Effects of clonality on the genetic variability of rare, insular species: the case of *Ruta microcarpa* from the Canary Islands. Ecol Evol.

[16] Tatarenko IV, Vakhrameeva MG (1998). Vegetative propagation in orchids (in Russian). Bulletin Botanicheskogo Sada imeni I.S. Kosenko Kubanskogo Gosagrouniversiteta.

[17] Gifford E, Foster A (1989). Morphology and Evolution of Vascular Plants.

[18] Arditti J (1979). Aspects of the physiology of orchids.

[19] Pellegrino G, Musacchio A, Noce ME, Palermo AM, Widmer A (2005). Reproductive *versus* floral isolation among morphologically similar *Serapias* L. species (Orchidaceae). J Hered.

[20] Baumann H, Künkele S (1989). Die Gattung *Serapias* L. - eine taxonomische übersicht. Mitteilungsblatt Beiträge zur Erhaltung Erforschung heimischer. Orchideen.

[21] D’Emerico S, Pignone D, Scrugli A (2000). Giemsa C-banded kary-otypes in *Serapias* L. (Orchidaceae). Bot J Linn Soc.

[22] Barone Lumaga MR, Pellegrino G, Bellusci F, Perrotta E, Perrotta I, Musacchio A (2012). Comparative floral micromorphology in four sympatric species of *Serapias* (Orchidaceae). Bot J Linn Soc.

[23] Delforge P (2006). Orchids of Europe.

[24] Bellusci F, Pellegrino G, Palermo AM, Musacchio A (2008). Phylogenetic relationships in the orchid genus *Serapias* L. based on non-coding regions of the chloroplast genome. Mol Phylogenet Evol.

[25] Vereecken NJ, Dafni A, Cozzolino S (2010). Pollination syndromes in Mediterranean orchids-implication for speciation, taxonomy and conservation. Bot Rev.

[26] Jersáková J, Johnson SD, Kindlmann P (2006). Mechanisms and evolution of deceptive pollination in orchids. Biol Rev.

[27] Schiestl FP, Cozzolino S (2008). Evolution of sexual mimicry in the Orchidinae: the role of preadaptations in the attraction of male bees as pollinators. BMC Evol Biol.

[28] Pellegrino G, Luca A, Bellusci F, Musacchio A (2012). Comparative analysis of floral scents in four sympatric species of *Serapias* L. (Orchidaceae): clues on their pollination strategies. Plant Syst Evol.

[29] Doyle JJ, Doyle JL (1987). A rapid DNA isolation procedure for small quantities of fresh leaf tissue. Phytochem Bull.

[30] Pellegrino G, Cafasso D, Widmer A, Soliva M, Musacchio A, Cozzolino S (2001). Isolation and characterization of microsatellite loci from the orchid *Serapias vomeracea* (Orchidaceae) and cross-priming to other *Serapias* species. Mol Ecol Notes.

[31] Valièr N (2002). GIMLET: a computer program for analysing genetic individual identification data. Mol Ecol Notes.

[32] Halkett F, Simon JC, Balloux F (2005). Tackling the population genetics of clonal and partially clonal organisms. Trends Ecol Evol.

[33] Pielou EC (1969). An introduction to mathematical ecology.

[34] Kalinowski ST (2004). Counting alleles with rarefaction: Private alleles and hierarchical sampling designs. Conserv Genet.

[35] Nei M (2013). Mutation-Driven Evolution.

[36] Goudet J (1995). FSTAT (Version 1.2): A Computer Program to Calculate F-Statistics. J Hered.

[37] Rousset F, Balding DJ, Bishop M, Cannings C (2007). Inferences from spatial population genetics. Handbook of statistical genetics.

[38] Weir BS, Cockerham CC (1984). Estimating F-Statistics for the analysis of population structure. Evolution.

[39] Cornuet JM, Luikart G (1997). Description and power analysis of two tests for detecting recent population bottlenecks from allele frequency data. Genetics.

[40] Piry S, Luikart G, Cornuet JM (1999). BOTTLENECK: a computer program for detecting recent reductions in the effective population size using allele frequency data. J Hered.

[41] Marshall TC, Slate J, Kruuk LEB, Pemberton JM (1998). Statistical confidence for likelihood-based paternity inference in natural populations. Mol Ecol.

[42] Luikart G, Sherwin WB, Steele BM, Allendorf FW (1998). Usefulness of molecular markers for detecting population bottlenecks via monitoring genetic change. Mol Ecol.

[43] Pellegrino G, Palermo AM, Noce ME, Bellusci F, Musacchio A (2007). Genetic population structure in the Mediterranean *Serapias vomeracea*, a nonrewarding orchid group. Interplay of pollination strategy and stochastic forces?. Plant Syst Evol.

[44] Hedrén M, Nordström S, Ståhlberg D (2012). Geographical variation and systematics of the tetraploid marsh orchid *Dactylorhiza majalis* subsp. *sphagnicola* (Orchidaceae) and closely related taxa. Bot J Linn Soc.

[45] Gustafsson S, Lönn M (2003). Genetic differentiation and habitat preference of flowering-time variants within *Gymnadenia conopsea*. Heredity.

[46] Stark C, Michalski SG, Babik W, Winterfeld G, Durka W (2011). Strong genetic differentiation between *Gymnadenia conopsea* and *G. densiflora* despite morphological similarity. Plant Syst Evol.

[47] Soliva M, Widmer A (2003). Gene flow across species boundaries in sympatric, sexually deceptive *Ophrys* (Orchidaceae) species. Evolution.

[48] Mant J, Peakall R, Schiestl FP (2005). Does selection on floral odor promote differentiation among populations and species of the sexually deceptive orchid genus *Ophrys*?. Evolution.

[49] Swarts ND, Sinclair EA, Krauss SL, Dixon KW (2009). Genetic diversity in fragmented populations of the critically endangered spider orchid *Caladenia huegelii*: implications for conservation. Conserv Genet.

[50] Chen YY, Bao Z-X, Qu Y, Li W, Li Z-Z (2014). Genetic diversity and population structure of the medicinal orchid *Gastrodia elata* revealed by microsatellite analysis. Biochem Syst Ecol.

[51] Abeli T, Gentili R, Mondoni A, Orsenigo S, Rossi G (2014). Effects of marginality on plant population performance. J Biogeogr.

[52] Leimu R, Mutikainen P, Koricheva J, Fischer M (2006). How general are positive relationships between plant population size, fitness and genetic variation?. J Ecol.

[53] Palstra FP, Ruzzante DE (2008). Genetic estimates of contemporary effective population size: what can they tell us about the importance of genetic stochasticity for wild population persistence?. Mol Ecol.

[54] Davies SJ, Cavers S, Finegan B, White A, Breed MF, Lowe AJ. Pollen flow in fragmented landscapes maintains genetic diversity following stand-replacing disturbance in a neotropical pioneer tree, *Vochysia ferruginea* Mart. Heredity. 2013; 1–5. 10.1038/hdy.2013.95 PMC4815448 24105437

[55] Lind H, Franzén M, Pettersson B, Nilsson LA (2007). Metapopulation pollination in the deceptive orchid *Anacamptis pyramidalis*. Nord J Bot.

[56] Mustajärvi K, Siikamäki P, Rytkönen S, Lammi A (2001). Consequences of plant population size and density for plant–pollinator interactions and plant performance. J Ecol.

[57] Setsuko S, Nagamitsu T, Tomaru N (2013). Pollen flow and effects of population structure on selfing rates and female and male reproductive success in fragmented *Magnolia stellata* populations. BMC Biol.

[58] Aguilar R, Ashworth L, Galetto L, Aizen MA (2006). Plant reproductive susceptibility to habitat fragmentation: review and synthesis through a meta-analysis. Ecol Lett.

[59] Vandewoestijne S, Róis AS, Caperta A, Baguette M, Tyteca D (2009). Effects of individual and population parameters on reproductive success in three sexually deceptive orchid species. Plant Biol.

[60] Schiestl FP (2004). Floral evolution and pollinator mate choice in a sexually deceptive orchid. J Evol Biol.

[61] Wong BBM, Salzmann C, Schiestl FP (2004). Pollinator attractiveness increases with distance from flowering orchids. Proc R Soc Lond.

[62] Brzosko E, Wroblewska A, Ratkiewicz M (2002). Spatial genetic structure and clonal diversity of island populations of lady’s slipper (*Cypripedium calceolus*) from the Biebrza National Park (northeast Poland). Mol Ecol.

[63] Brzosko E, Talalaj I, Wroblewska A (2006). Genetic structure of rare *Epipactis atrorubens* populations from two national parks in Northeast Poland. Pol Bot St.

[64] Brzosko E, Wroblewska A (2003). Genetic variation and clonal diversity in island *Cephalanthera rubra* populations from the Biebrza National Park. Poland Bot J Linn Soc.

[65] Silvertown JW (2008). The evolutionary maintenance of sexual reproduction: evidence from the ecological distribution of asexual reproduction in clonal plants. Int J Plant Sci.

[66] Vallejo-Marín M, Dorken ME, Barrett SCH (2010). The ecological and evolutionary consequences of clonality for plants mating. Annu Rev Ecol Syst.

[67] Lhuillier E, Butaud JF, Bouvet JM (2006). Extensive clonality and strong differentiation in the insular Pacific tree Santalum insulare: implications for its conservation. Ann Bot.

